# An Analysis of Cases of Paronychia

**Published:** 1855-10

**Authors:** Austin Flint

**Affiliations:** Buffalo, N. Y.


### BUFFALO MEDICAL JOURNAL

AND

### MONTHLY REVIEW.

**VOL. 11.** OCTOBER, 1855. **NO. 5.**

### ORIGINAL COMMUNICATIONS.

ART. I.—*An Analysis of Cases of Paronychia.*
By Austin Flint, Jr., Buffalo, N. Y.

### PARONYCHIA BENIGNA.

The private records of Dr. F. H. Hamilton, contain eighty-one cases of paronychia benigna.

In all these cases the sex has been noted; and we find the proportion of females to males, nearly two to one: there being fifty-three females and twenty-eight males. When we consider the occupation of these patients, and, as far as we can ascertain, the causes which have operated to produce this affection, we will see, that in the great majority of cases, their occupation involves manual labor, and where the occupation has been noted as the cause of the disease, it is most frequently an occupation in which females are generally engaged. In the male patients, the cause has generally been a bruise or slight injury to one of the fingers, and not the occupation. As female duties frequently cause paronychia, and accidental injuries are the most frequent causes in the male subject, we are not surprised to find a large proportion of cases occurring in females.

In sixty-one cases, the hand, affected with the paronychia, was designated.

Of these cases, forty-two occurred on the right hand, and nineteen on the left; showing that the right hand being more used and more subject to accidental injuries, is much more liable to this affection.

In seventy-five cases, the diseased finger has been designated. The thumb and first finger, which are most used, are affected in the greatest proportion of cases, and appear to be about equally liable to the disease. The thumb was the seat of the affection in thirty-two cases, and the first finger in twenty-seven. The second finger is next in order, being affected in ten cases. There were but two cases affecting the third finger; one affecting the fourth finger; two affecting the first, second and third; and one affecting the second and third. The last three cases were of the first variety, onychia cutanea; one of them was caused by pricking the finger in sewing: this was very trivial, and got well spontaneously. The other two were caused by suppressed menses, and were soon cured by argenti nitras and poultice. Thus, not only the hand, but the fingers which are most used, are most liable to paronychia.

In sixty-three cases, the age of the patient was noted.

I find but three cases under fifteen years of age. One of these patients was two years of age, another three years, and another eleven years. There are thirty-eight cases from fifteen years to thirty; nineteen from thirty to forty-five; two from forty-five to sixty, (one aged forty-eight and the other fifty); and one from sixty to seventy-five, (aged sixty-one).

According to these facts, paronychia is rarely met with in patients under fifteen years of age, or over forty-five; though it may appear at the age of two or three years, or as late as sixty-one. The favorite age appears to be from fifteen to thirty, at which period nearly two-thirds of the cases, where the age has been recorded, have occurred. It is by no means infrequent, however, from thirty to forty-five, at which period nearly one-third of the cases have occurred.

In sixty-three cases the occupation of the patient has been recorded.

All of these cases, but seven, occurred in persons engaged in occupations requiring considerable manual labor. These seven exceptions were, a clerk, a clergyman, a doctor, a student of medicine, a female teacher, and two children at school. The remaining fifty-seven were mechanics, laborers, and housemaids. Among them were twenty-three housemaids, five housekeepers,*

* By housekeeper is meant, not a servant, but the lady of a house.

five seamstresses, and two laundresses. Among the males were five shoemakers, four laborers, two sailors, two blacksmiths, etc., etc.

By far the largest number of cases, occurring in patients of any one occupation, is the number of housemaids, there being twenty-three out of sixty-three: more than one-third of the entire number, and nearly five times the number of cases of any other occupation, five being the next highest number.

In fifty-one cases the probable cause of the affection is recorded.

Under this head, I find the occupation recorded as the cause, in twenty-three cases. Of these, nine were accustomed to housework, scrubbing, etc. One of the patients, a shoemaker, says that shoemakers are particularly liable to paronychia, from their turning boots inside out. A similar statement was made by another patient, a lather, who said that lathers were liable to it, from driving small nails and frequently bruising their fingers. We also have a whip maker, and a sailor, in this number.

Five cases were caused by bad health; two cases were the result of slight wounds; two cases were caused by suppressed menses. These cases were of the first variety, onychia cutanea, and were treated with nitrate of silver and poultice. One case (a boy, three years old) was caused by his being deprived of meat, under the impression that he would thus avoid the cholera. He had been accustomed to meat diet, and was ordinarily healthy, but now has a paronychia and cankers in his mouth and nose.

A clergyman brought on a felon, by rowing a boat; a school girl, by practicing on the piano; and a housemaid, by washing dishes. One case occurred after an attack of ship fever.

Another case (P—— M——, a seamstress,) occurred in a patient of a scrofulous diathesis, who had an anal fistule.

From the foregoing facts we see that the causes of paronychia are various. The most frequent causes, however, are exposure of the hands to hot water, as in washing dishes, or a slight hurt or bruise. In forty-one cases the causes were of this description. More rarely the cause is general or constitutional, though this is sometimes the case. In ten cases the cause has been recorded, bad health, ship fever, suppressed menses, etc.

Of the entire number of cases, one was not properly a paronychia, but was a fungus at the root of the nail; eight were of the first variety, onychia cutanea; five were of the first and second variety, cutanea and cellulosa; and the remaining sixty-seven were of second, third, and fourth varieties, onychia cellulosa, tendonosa, and osteosa.

In the case of the fungus at the root of the nail, nothing is recorded, but that on the twenty-first day the fungus was the size of a pea.

The cause was recorded as constitutional in three of the five cases, which were of the first and second variety combined; there was no record, under this head, in the remaining two cases. In three of these cases the treatment was merely the application of arg. nit.; in one case it opened spontaneously on the eighth day, but the inflammation extended to the areolar tissue, pus was formed, and it was opened on the fourteenth day; in the remaining case there is no record under the head of treatment.

All these cases resulted in perfect cures.

Of the eight cases of the first variety, onychia cutanea, six were caused by slight injuries, and two were dependent upon constitutional causes. Six were treated with arg. nit, and poultices; one with a poultice alone; and one got well spontaneously. One, however, was opened before the arg. nit. was applied.

Four of these cases resulted in a perfect cure: one of them in the loss of the nail, and the remaining three appear to have been lost sight of.

In the sixty-seven cases which were of the second, third, and fourth varieties, I shall consider only one feature in the treatment, namely, whether it was opened or not, and how long after the commencement of the disease before it was opened. The other measures of treatment have been so imperfectly recorded, that their consideration would be of little value.

In fifty-five cases the result has been recorded. Thirty-six resulted in perfect cures; and nineteen, either in loss of part of the finger, or anchylosis.

Of the thirty-six cases, twenty-nine were opened; in the remaining seven cases, there is no record under this head. In twenty-seven cases pus had been formed; the remaining nine did not suppurate.

Of the twenty-nine cases where the finger was laid open, ten were opened on the seventh day: eight of these had suppurated and two had not. Seven were opened on the tenth day; three on the fourteenth day; two on the thirteenth day; one on the eighth day; one on the eleventh day; and one on the fifth day. All of these had suppurated. Two were opened on the fourth day, one having suppurated and one not. One was opened on the third day, and one on the second day: neither of them having suppurated.

In the nineteen cases where the cure was imperfect, twelve were opened, and in seven there was no record under this head.

One was opened on the twenty-eighth day; one on the twentieth day; one on the fifteenth day; one on the fourteenth; two on the seventh; and one on the fourth day. In the last case no pus was found. One cas opened spontaneously. This case was treated by an empiric, who was prosecuted and mulcted in damages, for not opening the finger. The case resulted in a loss of the entire finger.

From the results of these fifty-five cases, we see that we may expect a perfect cure in the majority of cases. In the cases which resulted in perfect cures, only six, out of twenty-nine, were opened after the tenth day; seven were opened on the tenth day, and ten on the seventh day; and one was opened as early as the second day, before pus had been formed. None were opened later than the fourteenth day. But in the cases where the cure was imperfect, one was not opened at all; seven were opened from the fourteenth to the twenty-eighth day; two only were opened as early as the seventh day, and one on the fourth day.

These facts show the importance of opening the finger at least as early as the tenth day, and it would appear to be proper to do so, as soon as the disease has become established; even before pus has been formed.

### INFLAMMATION OF THE FINGER OF THE SAME CHARACTER AS PARONYCHIA, BUT NOT IN THE LAST PHALANX.

Dr. Hamilton has recorded eighteen cases which came under this head. We will see, that in nearly every particular, they are the same as paronychia proper; but as there are quite a number of these cases, it may be as well to consider them by themselves.

As in paronychia, female patients predominate: ten being females, and eight males. The age which seem most liable to the affection, is also the same. No case occurred under fifteen years; twelve cases from fifteen to thirty; three from thirty to forty-five, two at thirty-two years, and one at thirty-seven. No cases occurred after the age of thirty-seven.

The right hand was affected in eight out of ten cases, where this was noted. Out of thirteen cases the thumb was affected in one case; the first finger in one case; the second finger in six cases; the third finger in five cases; but in no case was the fourth finger affected. Here we see a difference from the same part in paronychia, where the thumb and first finger were affected in a great majority of cases. I can see no cause for this difference, excepting, perhaps, that the causes which produce this affection appear to be more purely accidental than in paronychia, and not so much due to the occupation of the patient; and that in reality it is the last phalanges of the thumb and forefinger which are most used, not the entire finger and thumb, and the other phalanges of the second and third fingers are, perhaps, more liable to accidental injuries, than the first phalanx of the thumb, or the first or second phalanges of the fore-finger.

Of sixteen cases, the first phalanx is affected in four cases; the first and second, in one case; the second, in five cases; the second and third, in one case; the metacarpo-phalangeal articulation, in five cases. This shows that the first and second phalanges are about equally liable to it; and also the metacarpo-phalangeal articulation.

As regards the occupation of the patients we will see that the same classes of society are affected with this disease as those affected with paronychia. Here, also, housemaids and housekeepers predominate. We have, of seventeen cases, six housemaids, three housekeepers, two sailors, two stone cutters, one cabinet maker, one male cook, one cooper, and one clergyman. The clergyman is the same referred to in the cases of paronychia: it occurred at the same time with the paronychia, and was induced by the same cause, namely, rowing a boat.

As regards the causes which operated to produce the disease, of ten cases where the cause was noted, in five the occupation was recorded as the cause. These five cases embraced four housemaids and one cabinet maker. In one case the cause was a slight cut; in another, a slight burn; in another, a slight bruise; and another was caused by rowing a boat.

Fourteen cases were recorded as having been opened: two, eight days after the commencement of the disease; five, seven days after; six, five days after; and one, three days after.

All had suppurated, where this point was noted, excepting one case.

Ten cases were recorded as resulting in perfect cures; and one as resulting in a permanent contraction of the finger. This last case was opened on the seventh day, but subsequently became very much inflamed and suppurated profusely.

This affection differs from paronychia, only in the situation of the inflammation; it is of precisely the same character, and, of course, demands the same treatment, namely, where the inflammation is not superficial, an early and free opening. It appears, however, to be rather less formidable than paronychia, and that the result is more generally favorable. In only one of eleven cases of inflammation of the first or second phalanges, was the cure imperfect, and in this case the inflammation ran very high after the finger was opened, and resulted in permanent contraction of the flexors; while in nineteen out of fifty-five cases of paronychia proper, the cure was imperfect, and in some of these cases the last phalanx, or even more of the finger, was lost.

Dr. Hamilton informs me, however, that in two or three cases, not recorded, he has seen an entire phalanx destroyed by necrosis.

### PARONYCHIA MALIGNA.

I have records of seven cases of paronychia maligna. Six of these cases were recorded by Dr. Hamilton, and one case I recorded at the clinical lectures by Prof. Gross, of the Louisville University.

Case I. Joseph Carnin, aged nine years; admitted to the Buffalo Hospital of the Sisters of Charity, Oct. 19, 1848. Habit scrofulous; the extremity of the great toe of the left foot was prodigiously enlarged, red, and slightly tender; the nail was black, rotten as pasteboard, and stood directly up from the matrix. This has been his condition for about a year.

A bread and water poultice was applied the first twenty-four hours, and from this time ung. hyd. rub. or the ung. hyd. nit. were applied daily to the foot. The diet was generous, and he is allowed occasional exercise. Under this treatment the malady gradually disappeared, the cure being accomplished in about three months.

Case II. Geo. Vangu, aged seven years. He had scarlatina six months since, and was very ill, but now looks healthy. The onychia commenced soon after his recovery from scarlatina. Nail is black and rotten; the affection is seated on the second finger of the right hand. Cause, constitutional and local. Treatment has been poultices, unguents, caustics, etc., etc. Corrosive sublimate wash increased the irritation; poultices gave most relief. Result is unknown.

Case III. Thomas O'Connor, aged six years. Five weeks ago he split the nail of his third finger; one week since he bruised it. It has been treated with poultices. The nail became black and fell off, but was reproduced, and of the same character as before, black and rotten. In this condition the finger remained many months, under a variety of plans of treatment. Soothing poultices gave most relief, especially when combined with tonics and outdoor exercise. It was eventually cured.

Case IV. Henry W. Putnam, aged nine years. Disease affects the thumb. Cause, constitutional and local. As regards treatment, almost every thing which has been recommended, was tried. Six nails have fallen off successively since the disease began. End of thumb is flat, sides puffed out and of a purplish red color; lower half of nail black and rotten; ulceration under the nail; the parts in the vicinity are red and puffy. The result of this case was a cure.

Case V. Williams, male, aged five years. Cause, probably constitutional. He was apparently in good health, but had slight eruptions, on various parts of his body. It has been treated by caustics, arsenic, corrosive sublimate, poultices, tonics, hyd. chlor. mite., etc., etc. The ulceration did not cease when the matrix of the nail was gone. Ung. nit. in substance, was first applied, but this only increased the irritation; hyd. chlo. corros. was then applied with the same result. The end of the thumb was then shaved off, but the ulceration attacked the stump, and it was finally necessary to amputate the last phalanx, when the wound slowly healed.

Case VI. Wm. Fitzgerald, aged eight years. He has onychia maligna affecting the second finger of the right hand. Cause, constitutional and local; he projected a sliver under the nail four months since. It has been poulticed occasionally. The finger presents the usual appearance, excepting that the nail is not blackened. He has scrofulous ulceration; his mother is scrofulous. The poultices, with good diet and cleanliness, effected most. This case was cured after several months.

Case VII. Catharine Hynes, aged nine years; has onychia maligna affecting the great toe of the left foot. Cause, constitutional and local. She has a scrofulous appearance and received a severe contusion upon the toe. Nail was removed by Prof. Gross, and the toe has the characteristic shovel shaped appearance. Treatment was the local application of blue wash, and slight mercurialization.

I saw the case some days after and it was progressing favorably.

This case I saw at the Louisville Marine Hospital, in 1854; it was treated by Dr. Gross.

Paronychia maligna is, according to our observation, a disease peculiar to children. In the foregoing cases we have none over nine years of age. The favorite age appears to be from eight to nine. We have three cases nine years of age, and one case eight years. We also have one case seven years; one case six years; and one case five years of age. There are no cases under five years.

All the cases recorded by Dr. Hamilton were males; and the single case which was seen by myself, was a female. This shows a very great predominence of males.

The affected hand or foot has not been noted in a sufficient number of cases to make its consideration of value.

The great toe was affected in two cases; the thumb in two cases; the second finger in two cases; and the third finger in one case. Thus we see that the hand is affected in five cases, and the foot in only two, showing that the affection is much more frequently seated in the finger or thumb than in the great toe, which I believe is contrary to the general opinion. Where the foot is affected, it has always attacked the great toe; but in the cases where the hand was affected, it has attacked the thumb or second and third finger. No cases are recorded of the disease seated in the first or fourth finger.

In all these cases but one, the cause appears to be either purely constitutional, or at least partially so. In one case, however, the cause was apparently local, (Case, No. 4, H. W. Putnam,) though the progress of the disease, six nails falling off since the commencement, shows that the system was somewhat at fault. In three cases the cause was purely constitutional; and in three cases it was constitutional and local.

Such a variety of treatment has been practiced in these cases, that its consideration with reference to the result, is of little value. Tonics, cleanliness, and soothing applications, appear to have had the best effect; and mild mercurial applications have been used with advantage in two cases, in connection with outdoor exercise, tonics and poultices. Caustics, where they have been used, have not been productive of any good effects; they appear rather to have aggravated the disease.

Four cases, Nos. 1, 3, 4 and 6, are known to have resulted in perfect cures. In three cases tonic measures and poultices were used. In addition to these measures, in one case, ung. hyd. rub. dilutum, was used. In one case, No. 5, it was necessary to amputate the last phalanx of the thumb. In one case, No. 2, the result was unknown. I saw case, No. 7, several times at the Louisville Marine Hospital; it was progressing favorably, and probably resulted in a perfect cure.

From these facts we would infer, that although the disease is likely to be protracted and tedious, yet with proper care we may expect a perfect cure. Amputation will rarely be necessary.

